# *In Vitro* Assessment of Biological and Functional Properties of Potential Probiotic Strains Isolated from Commercial and Dairy Sources

**DOI:** 10.3390/microorganisms13050970

**Published:** 2025-04-24

**Authors:** Elmira Kelidkazeran, Meriam Bouri Yildiz, Fikrettin Sahin

**Affiliations:** Faculty of Engineering, Department of Genetics and Bioengineering, Yeditepe University, 34755 Istanbul, Türkiye; elmira.kelidkazeran@yeditepe.edu.tr (E.K.); meriam.yildiz@yeditepe.edu.tr (M.B.Y.)

**Keywords:** probiotics, enzymatic activity, antioxidant activity, DPPH scavenging, antimicrobial effects, antibiotic resistance, gastrointestinal tolerance

## Abstract

Probiotic species have garnered significant attention for their health benefits extending beyond gastrointestinal health. This study investigated the biological and enzymatic functions of selected probiotic species, specifically *Lacticaseibacillus rhamnosus* (formerly *Lactobacillus rhamnosus*), *Lactiplantibacillus plantarum* (formerly *Lactobacillus plantarum*), *Lactobacillus acidophilus*, and *Lactobacillus delbrueckii*, among others, through *in vitro* experiments. Enzymatic activities, including hemolytic, lipase, esterase, and protease functions, were evaluated. Antioxidant capacity was assessed using DPPH radical scavenging assays, while antimicrobial efficacy was tested against common pathogenic bacteria. Antibiotic-resistance patterns were analyzed to ascertain their safety for human consumption. Furthermore, simulated digestive fluid tolerance experiments were conducted to evaluate survival in the gastrointestinal tract. The findings indicate that these probiotic strains exhibit diverse functionalities beyond intestinal health, with potential roles in digestion, oxidative stress reduction, and immune support. This study provides valuable insights into the functional diversity of probiotics, suggesting their broader applications in health and nutrition. Future research should focus on *in vivo* validation, mechanism elucidation, and clinical studies to determine optimal dosages and strain-specific benefits.

## 1. Introduction

In recent years, there has been growing interest in the role of microorganisms in human health, particularly in the context of probiotics. Probiotics represent a unique category of organisms that actively contribute to human health through their living presence in the body [1,2]. The Food and Agriculture Organization (FAO) and World Health Organization (WHO) define probiotics as “live microorganisms which, when administered in adequate amounts, confer a health benefit on the host” [3]. These microorganisms play a crucial role in maintaining gut equilibrium, enhancing nutrient absorption, modulating the immune system, and influencing mental well-being through the gut–brain axis [4,5]. Research suggests that probiotics may also improve skin health, assist in weight management, and reduce the risk of chronic diseases [6]. Among probiotic bacteria, lactic acid bacteria have been extensively studied due to their lactic acid production and antimicrobial characteristics. Well-documented strains such as *Lacticaseibacillus rhamnosus* GG (formerly *Lactobacillus rhamnosus* GG), *Lactobacillus acidophilus*, and *Lactiplantibacillus plantarum* are frequently utilized in both pharmaceutical and food-based probiotic products [7]. In line with recent taxonomic updates, the genus *Lactobacillus* has been reclassified into 23 new genera [8]. Accordingly, we have updated all bacterial names throughout the manuscript to reflect this revised classification and ensure consistency with current nomenclature standards. This study aimed to investigate the biological characteristics of selected strains with established clinical backgrounds versus naturally occurring strains in fermented food. Furthermore, *Leuconostoc mesenteroides* and *Enterococcus faecium* have garnered attention for their potential to improve gut microbiota balance and relieve gastrointestinal problems [9].

While numerous commercial products assert probiotic benefits, it remains uncertain whether bacterial strains from diverse sources exhibit comparable biological characteristics [10]. In this study, some strains were extracted from pharmaceutical probiotic products and used as reference strains, while others were sourced from commonly consumed organic dairy items [11,12]. In recent years, there has been a growing interest in understanding the origins of probiotic strains, prompted by evidence indicating that bacterial isolates from diverse ecological and commercial sources may possess distinct functional attributes [13,14]. Recent comparative analyses have highlighted this variability, revealing that even within a single species, the biological behavior of strains can be significantly influenced by their environmental origins [15,16,17,18]. For instance, strains such as *L. rhamnosus*, *L. paracasei*, and *L. plantarum* have demonstrated variations in their tolerance to acid and bile, antimicrobial properties, and enzymatic activities, contingent upon their isolation from human microbiota, dairy products, or pharmaceutical supplements [8,15,16,17]. In addition, the survival of probiotic strains might differ depending on their matrix, with some strains potentially benefiting from the buffering effects of fermented dairy products, thus surviving acidic conditions in the stomach. This understanding is vital for optimizing strain selection in the development of probiotic products and their clinical use. This comparative analysis is crucial for enhancing our comprehension of how the ecological origins of probiotic strains may affect their functional characteristics. By investigating strains derived from both organically fermented foods and clinically validated supplements, this study sought to strengthen the scientific foundation for the selection, standardization, and application of probiotics in both nutritional and therapeutic domains.

The gastrointestinal tract, particularly the stomach, presents a significant challenge to the survival of probiotic bacteria because of its highly acidic environment (pH 1.5–3.5) and the presence of digestive enzymes such as pepsin [19]. In this study, we sought to determine the resilience of different commercial probiotic strains under conditions that mimic the gastric environment using a detailed *in vitro* experimental method. The experimental methodology involved subjecting the bacteria to simulated gastric fluid (pH 2.0, containing pepsin) for a duration of two hours at 37 °C, replicating the conditions within the human stomach. Bacterial viability was assessed by enumerating colony-forming units, yielding survival profiles for each strain under examination [20]. This experimental approach provides valuable insights into the capacity of probiotic strains to withstand gastric conditions, which is essential for developing efficacious probiotic formulations.

Research has demonstrated that probiotic strains may exhibit enzymatic activities that contribute to various physiological processes in the human body [21]. This study focused on three pivotal enzymes produced by these strains—lipases, esterases, and proteases—which are commonly associated with digestive and metabolic support. Strains exhibiting lipase activity are integral to lipid metabolism, facilitating fat digestion, enhancing the production of beneficial metabolites, improving nutrient absorption, and potentially offering anti-inflammatory benefits to the host [22]. Evidence suggests that certain lipase-producing probiotic strains can lower cholesterol levels by breaking down cholesterol esters and may aid in alleviating symptoms associated with pancreatic insufficiency or fat malabsorption [23]. It is important to note that the enzyme activity assessments presented in this study were conducted via *in vitro* plate-based assays. These methods provide preliminary indications of enzymatic potential, and further investigations involving functional and clinical validation are necessary to substantiate these findings.

Probiotic strains with esterase activity have been found to break down complex food components, release bioactive compounds from plant-based foods, and affect the composition of the gut microbiota [23]. Conversely, protease activity aids in protein digestion, producing bioactive peptides that may offer health benefits and potentially decrease the allergenic properties of certain proteins [24,25]. Bacterial strains possessing these enzymatic activities offer a range of health benefits and can improve the nutritional quality and functionality of products [26].

This study aimed to elucidate the acid tolerance and survival capabilities of various pharmaceutical and fermented commercial probiotic strains. We conducted comprehensive *in vitro* analysis to assess the enzymatic activities, antimicrobial resistance, and antibiotic susceptibility of these potential bacterial strains. By evaluating these key characteristics, we aimed to determine whether probiotics of pharmaceutical and food origins exhibit similar properties and to identify strains that may be more suitable for specific applications. Our findings provide valuable insights into the selection and optimization of probiotics for both commercial and therapeutic uses, contributing to the advancement of probiotic research and development. The results of this study have important implications for the formulation of probiotic products and the potential tailoring of strains for targeted health benefits. Although these findings provide valuable insights into the functional properties of these strains, further *in vivo* and clinical studies are necessary to confirm their health benefits.

## 2. Materials and Methods

### 2.1. Commercial Products

Seventeen commercial products containing probiotic bacteria were obtained from various sources (Table 1). A batch of five samples was analyzed for each product. Product conformity was evaluated in relation to the species and CFU/mL or CFU/g information provided in the packaging.

### 2.2. Bacteria Isolation and Identification

Samples from the free-flowing powders of microencapsulated cell cultures, tablets, and capsules were processed according to the recommendations of Champagne et al. [27]. Fresh products were utilized in the analysis, and no freeze–thaw cycles were applied. Dehydration and dissolution were conducted using an MRD (maximum recovery diluent) solution (1.46809 Merck, Darmstadt, Germany). Serial dilutions were prepared in MRD solution and plated on MRS (Mann-Rogosa-Sharpe) agar medium (110660 Merck, Darmstadt, Germany). The plates were prepared in duplicates and incubated at 37 °C for 48 h. For anaerobic species, a 5% CO_2_ incubator was used. After purification, the bacterial species were identified using Maldi-TOF (Bruker Daltonics, Bremen, Germany) following the manufacturer’s protocol. Identification scores ≥ 2.0 were considered reliable at the species level.

To uphold ethical standards and scientific neutrality, all probiotic products were anonymized using internal codes (e.g., P1–P5 for supplements and F1–F10 for food products). Product selection was based on the presence of clinically validated probiotic strains, and only strains that were consistently isolated across triplicate samplings were included in the final analysis. All strain IDs (e.g., M2, M9, M11, …) represent internally coded isolates for traceability within this study and do not reflect any official nomenclature or product-specific labeling.

### 2.3. Acid and Bile Resistance Assay

Selected strains were subjected to MRS broth (M369-500G Himedia, Mumbai, India) at pH 2.0, 2.5, and 3.0, and MRS broth with dehydrated fresh bile 0.3%, 1.0%, an 2.0%, (*w*/*v*) to evaluate their acid and bile resistance, respectively [28]. MRS broths were adjusted with 1 M HCl or Oxgall (dehydrated fresh bile) (LP0055 Oxoid, North Shore City, New Zealand) and inoculated with 1% (*v*/*v*) 0.5 McFarland overnight suspensions of the tested strains. Control samples of MRS broth without bacterial inoculation and blank samples without bacterial inoculation were prepared. The samples were incubated at 37 °C for 3 h, either aerobically or anaerobically, depending on the strain. Absorbance at 560 nm was measured hourly to track growth, and the survival rate was calculated using the formula:

$$\mathrm{Survival}\;\mathrm{Rate}\;(\%)=(\frac{FinalAbsorbance}{Initial\;Absorbance}\times 100)$$

In cases where absorbance did not correlate with growth, plate counts were also performed to confirm strain viability.

### 2.4. Antibiotic-Susceptibility Assay

The antibiotic resistance of the isolated probiotic bacteria was assessed according to the Kirby–Bauer disk diffusion susceptibility test protocol recommended by the Clinical and Laboratory Standards Institute [29]. A volume of 100 μL of overnight probiotic culture (10^8^ CFU/mL) was spread onto MRS agar. Disks impregnated with erythromycin (15 µg) (Oxoid, Basingstoke, UK), gentamycin (10 µg) (Oxoid, Basingstoke, UK), and clindamycin (2 µg) (Oxoid, Basingstoke, UK) were placed on agar. In this study, strains sourced from probiotic supplements were utilized as comparative benchmarks, and a blank disk inoculated with sterile water was used as a negative control to confirm that any inhibition zones were solely due to antibiotic activity and not external factors. The susceptibility of each strain was determined by comparing the inhibition zones to the National Committee for Clinical Laboratory Standards (NCCLS) guidelines, classifying strains as susceptible, intermediate, or resistant.

### 2.5. Antimicrobial Activity Assay

The agar double-layer method, as per Kamel et al., was employed to evaluate the antimicrobial activity of probiotic bacteria against a panel of pathogenic strains that are clinically significant and commonly associated with infections (Table 1) [30]. Briefly, 20 μL of the probiotic suspension (10^8^ CFU mL^−1^) in sterile distilled water was spot-inoculated onto MRS agar plates. After incubation under optimal conditions for 48 h, 50 μL of chloroform (34854 Sigma-Aldrich, Steinheim, Germany) was added to the probiotic strains and left for 15 min in a laminar flow cabinet. The pathogens (*E. coli*, *S. aureus*, *P. aeruginosa*) were grown separately in broth, and 1 mL of each pathogenic suspension (10^8^ CFU mL^−1^) of *E. coli*, *S. aureus*, and *P. aeruginosa* was combined with 3 mL of TSA (0.6% agar) (22091 Merck, Germany) at 45 °C and poured over the pre-inoculated probiotic strains on the MRS plates, and the plates were allowed to solidify. To ensure accuracy and comparability, a probiotic strain (*Lacticaseibacillus rhamnosus* GG) was used as a positive control to validate the antimicrobial activity of tested strains, and MRS agar plates without probiotic inoculation served as a negative control. After incubation at 37 °C, the plates were examined for the presence of inhibition zones.

### 2.6. Enzymatic Activity Assay

The enzymatic activities of the probiotic strains were evaluated by inoculating the strains onto several agar media: blood agar (PB5012 Thermo, Karlsruhe, Germany) for hemolysis, skim milk agar (115338 Merck, Darmstadt, Germany) for protease activity, MRS agar supplemented with 5% tributyrin (91015 Merck, Darmstadt, Germany) for lipase activity, and MRS agar supplemented with 10% Tween 80 (P1754 Sigma-Aldrich, St. Louis, MO, USA) for esterase activity [21]. Putative probiotic strains were inoculated onto agar plates and incubated for 48 h under optimal conditions. The presence of clear zones indicated positive protease and lipase activity. A positive result for esterase activity was detected based on the presence of visible precipitation around the colony (opaque halo). As for hemolysis tests, EFSA and FAO/WHO guidelines consider probiotic strains that show β-hemolysis to be unsafe, whereas those exhibiting α-hemolysis require additional evaluation to confirm the absence of virulence factors [1,31].

### 2.7. Intracellular Cell-Free Extract Antioxidant Activity Assay

Probiotic cell pellets were harvested by centrifugation at 12,000× *g* for 10 min at 4 °C and weighed. The cells were resuspended in phosphate buffer (0.2 mol/L, pH 7.1), and bacterial cells were disrupted for 15 min on ice using a sterile mortar and pestle to release bioactive compounds. The homogenized suspension was then centrifuged at 12,000× *g* for 10 min at 4 °C to remove cell debris, and the supernatant was collected. The supernatant was collected and subjected to a free radical scavenging assay to evaluate potential antioxidant activity. For the DPPH assay, 100 μL of the intracellular extract was mixed with 900 μL of freshly prepared 0.2 mM DPPH solution in methanol and incubated in the dark at room temperature for 30 min. The absorbance was measured at 517 nm using a UV-vis spectrophotometer following the method outlined by Rahmati et al. [32]. The antioxidant activity was calculated using the following formula:

$$\mathrm{DPPH}\;\mathrm{scavenging}\;\mathrm{activity}\;(\%)=(\frac{A0-A1}{A0}\times 100)$$

where A0 is the absorbance of the DPPH solution without extract (control) and A1 is the absorbance in the presence of the probiotic extract. Ascorbic acid (0.2 mM) was used as the positive control.

All experiments were conducted in triplicate, and results are expressed as means ± standard deviation.

### 2.8. Statistical Analysis

All experiments were conducted in triplicate. Data were analyzed using Microsoft Excel and GraphPad Prism 8.0.2. (263) was used to organize the data and create graphical representations of the results. One-way analysis of variance (ANOVA) was performed to determine significant differences among the treatments. Statistical significance was set at *p* < 0.05.

## 3. Results and Discussion

### 3.1. Probiotic Isolation, Enumeration, and Identification

As shown in Table 1, the analyzed samples exhibited varying cell densities and species. The bacterial cell density and identity of all the probiotic supplement brands were consistent with the manufacturer’s specifications. We limited the inclusion of strains to those that were isolated in consistent and sufficient quantities. This was particularly important to ensure that the strains used for comparison were comparable in their viability and probiotic potential. The focus remained on comparing strains isolated from organic dairy products, as the primary aim was to investigate potential differences in probiotic characteristics between food and supplement sources. Although no specific cell count level has been recognized to guarantee a health effect [33], the current recommended level for probiotic bacteria by the FAO/WHO is ≥10^6^ CFU/g of product [28,34]. In yogurt brands, bacterial cell density meets this minimum requirement. However, some studies on probiotic bacteria at higher [35] or lower [36] doses have been published. In cheese- and vegetable-fermented foods, bacterial densities were significantly lower and the plates were predominantly colonized by yeast rather than bacteria, particularly in cheddar cheese product. This can occur either through contamination or via spontaneous and commercial culture inoculation [37]. During early cheese ripening, the prevalence of yeast increases more rapidly than that of Gram-positive bacteria [38,39,40,41]. Yeast counts can reach 3.5 to 9 log CFU/mL in a brine solution [37,42,43,44,45].

For multispecies probiotic supplements, although the total density and all probiotic species conformed to the data specified on their packaging, the prevalence was not uniform. This disproportion in species frequency could be attributed to the manufacturing process (mass production, formulation, storage, etc.), strain incompatibility, or culture media artifacts. Generally, it is recommended that cell mass production be conducted separately to improve the quality of each strain in the final probiotic formulation [46]. Kailasapathy and Chin reported that some probiotic strains exhibit mutual inhibitory properties, thus necessitating special attention to consortium combinations for multispecies probiotics [47]. Moreover, differential and selective enumeration can be challenging when several species are simultaneously present in a product matrix and share similar cultural characteristics. Therefore, selective media can be considered for differential colony counts of each individual species. Although MRS medium is likely the most widely utilized base plating medium for pure cultures of lactic acid bacteria [32,48,49], Ashraf and Shah recommended the use of MRS agar supplemented with nalidixic acid, paromomycin, and neomycin sulfate, at 37 °C for 72 h [50].

While this study focused on dairy-derived probiotics, human-derived probiotics like *Lacticaseibacillus rhamnosus* and *Bifidobacterium bifidum* are commonly studied for their clinical applications in gut health and disease prevention.

### 3.2. Biological Characterization

Acid and bile resistance are essential for probiotics, as they enhance survival upon ingestion and transit to the gastrointestinal tract. Cell counts following acid and bile challenges were evaluated at various levels (Table 2).

All strains isolated from supplement products demonstrated resistance to different pH levels, exhibiting sensitivity to pH 2 and 2.5. Probiotics recovered from yogurt displayed low pH resistance (Table 2). Cheese-derived strains also demonstrated resistance to different pH levels, except for *L. delbrueckii* subsp. *bulgaricus*, which was unable to survive at pH < 3. *Leuconostoc mesenteroides* isolated from pickles was the only isolate that exhibited bile resistance and sensitivity to all pH levels. However, acid resistance did not always correlate with bile resistance. For instance, *L. acidophilus* and *L. plantarum* recovered from a probiotic supplement and pickles, respectively, were resistant to low pH, but not to any level of bile salt. This discrepancy could be attributed to differences between evaluation methods and assessed strains. Probiotic starter cultures of yogurt are generally described as sensitive to bile salts and acid [51].

These findings provide valuable information on the acid tolerance of probiotic strains, crucial for predicting potential efficacy *in vivo*. Strains with higher survival rates in gastric simulations may deliver sufficient quantities to the intestines to confer health benefits. *In vitro* results may not always translate directly to *in vivo* performance, and further studies are necessary to confirm potential therapeutic applications. The observed strain-specific survival rate underscores the importance of meticulous strain selection in probiotic product development and research.

The observation that probiotics isolated from probiotic supplements exhibit higher survival rates than those from fermented foods suggests commercial formulations may offer additional protection against stomach acid. This research emphasizes the potential for employing advanced formulation techniques to enhance probiotic efficacy and survivability. The identification of highly resistant lactic acid bacteria cultures maintaining over 80% viability after two hours of SGF exposure is particularly promising. These bacteria may serve as excellent probiotic candidates where prolonged stomach exposure is anticipated. Nevertheless, the substantial decline in viability observed in certain strains indicates that additional precautions or alternative delivery systems may be required to ensure survival in the digestive tract.

Antibiotic resistance is a critical factor for the assessment of probiotic strains. Some researchers assert that antibiotic resistance is often non-transmissible and that probiotics are essential for preserving healthy gut microbiota during antibiotic therapy [52], while others posit that antibiotic resistance is not transmissible in most cases and that probiotics are necessary to ensure healthy intestinal microbiota when antibiotic treatments are administered [52]. Consequently, in the safety assessment of probiotic candidates, antibiotic-resistant strains must not harbor resistance genes on mobile elements to prevent their transfer to other microorganisms. As represented in Table 3, most isolates exhibited susceptibility to the tested antibiotics, indicating a generally favorable safety profile. However, *Lactobacillus delbrueckii* subsp. *bulgaricus*, isolated from yogurt, demonstrated resistance to clindamycin and gentamicin. Additionally, some strains grew on erythromycin-containing agar, suggesting sensitivity to erythromycin despite initial indications.

**Figure 1:**
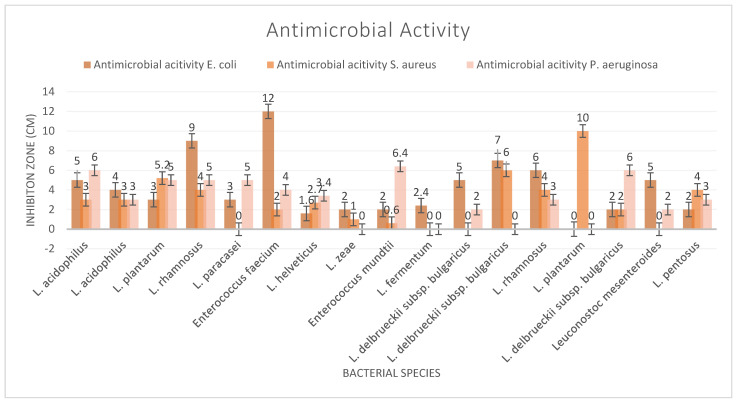
Antimicrobial activity test results.

Beneficial microorganisms, known as probiotics, show significant promise for their ability to combat harmful pathogens through various mechanisms. These include the production of organic acids and bacteriocins, competitive exclusion of detrimental bacteria, and stimulation of the host immune system, offering a comprehensive approach to suppressing pathogens. Supplements yielded two strains, *L. rhamnosus* and *E. faecium*, which showed significant results against *E. coli*, while the cheese-derived strain *L. plantarum* demonstrated a significant impact on *S. aureus* (Figure 1). The maximum inhibition zone of the bacterial strains against *P. aeruginosa* was 6.5 mm. The demonstrated effectiveness of probiotics against various pathogens suggests a potential shift in the development of novel probiotic-based therapies and products that offer alternatives.

**Figure 2:**
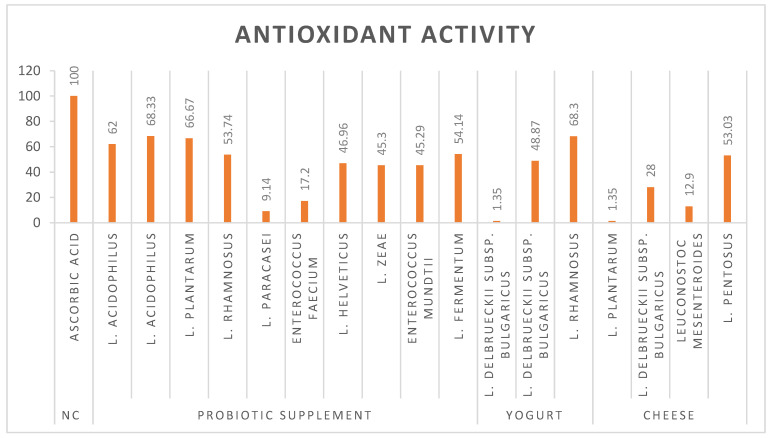
Antioxidant activity of bacterial strains.

*L. plantarum*, *L. rhamnosus*, and *L. acidophilus* have emerged as potent probiotic strains that demonstrate exceptional antioxidant activity. These potential probiotic strains demonstrate high efficacy (over 60%) in combating oxidative stress through free radical scavenging (Figure 2). The 2,2-diphenyl-1-picrylhydrazyl (DPPH) assay, a widely recognized method for evaluating antioxidant activity, has provided substantial evidence for the ability of probiotics to neutralize reactive oxygen species (ROS). The impressive antioxidant capabilities in these lactic acid bacteria have significant implications in both functional food development and therapeutic applications. Further research into the specific mechanisms of action and optimal dosages could pave the way for innovative probiotic-based interventions in both nutrition and medicine.

The enzymatic activities of the aforementioned bacterial strains were evaluated based on their clear zones after 48 h of incubation. Proteases are enzymes that catalyze the hydrolysis of proteins into smaller peptides or individual amino acids. In probiotic strains, protease activity may facilitate protein digestion and absorption in the intestinal system and alter the gut microenvironment by hydrolyzing proteins in the intestinal lumen. Moreover, probiotic strains potentially modulate immune responses by modifying protein-based signaling molecules. Accordingly, *L. acidophilus* and *L. rhamnosus* strains demonstrated protease activity irrespective of their isolation source. Although *L. plantarum* displayed a positive outcome when isolated from a single source, it failed to show any results when obtained from cheese.

Only two strains, *L. mesenteroides* and *E. faecium*, exhibited positive results for esterase and lipase activities. In probiotic strains, the ability to catalyze ester bond hydrolysis in various substrates may facilitate the decomposition of complex food components and potentially contribute to the metabolism of certain xenobiotics. Probiotics with lipase activity play a crucial role in breaking down triglycerides into fatty acids and glycerol, thereby improving fat digestion and absorption in the gastrointestinal tract. Additionally, these probiotics may alter the lipid composition of the intestinal environment, potentially influencing cholesterol metabolism.

Examination of hemolytic results suggests that probiotic strains are most desirable when they show no activity on blood agar, which is the case for most strains, as shown in (Table 4). Some strains displayed α-hemolysis, characterized by a green–brown discoloration surrounding colonies due to partial red blood cell lysis.

Evaluations indicate that certain strains may exhibit this phenomenon. Stojanov et al. (2024) conducted a study that further underscored the safety of lactic acid bacteria (LAB) strains for probiotic use, particularly emphasizing their non-toxic effects on human cells and the lack of hemolytic activity, thereby reinforcing the potential safety of these strains for human consumption [53]. However, further safety assessments, including virulence factors and antibiotic-resistance tests, are required for a more comprehensive investigation. None of the strains demonstrated β-hemolysis activity.

## 4. Conclusions

This study offers a comprehensive evaluation of bacterial strains isolated from both commercial supplements and dairy products, providing critical insights into the selection and formulation strategies for the development of probiotic products. The findings highlight the distinct survival patterns of each strain and offer insights into how different isolation sources influence the strains’ characteristics and functionalities, underscoring the necessity for thorough assessment prior to the incorporation of probiotics into various products.

Consistently with prior research, lactic acid bacteria demonstrated greater resistance to simulated gastric fluid (SGF) compared to other strains. Strains derived from supplements demonstrated enhanced gastric tolerance, with several lactic acid bacteria maintaining over 80% viability. These results underscore the critical role of formulation and strain specificity in the development of effective probiotic interventions.

Antimicrobial assays demonstrated that probiotics can inhibit *E. coli*, *S. aureus*, and *P. aeruginosa*; however, their effectiveness against antibiotic-resistant bacteria warrants further investigation. These findings suggest that potential probiotics may serve as an adjunctive strategy in the management of infectious diseases, underscoring the necessity for continued research in this area.

While some probiotic-potent strains exhibited robust antioxidant capacities, underscoring their potential to modulate immune responses and mitigate oxidative stress-related disorders. These findings emphasize the significance of strain origin and functionality in probiotic formulations.

Although these *in vitro* results are promising, further *in vivo* studies and clinical trials are essential to validate and support their application in clinical and functional food contexts.

## Figures and Tables

**Table 1:** Commercial products and isolated strains.

Product Code	Type	Total Plate Count ^a^	Identified Probiotic Species *	MALDI-TOF Reliability Score	Conformity ^b^
P1	Probiotic supplement	4.3 × 10^11^ CFU/g	*Lacticaseibacillus rhamnosus*	2.37	Yes
P2	Probiotic supplement	4.1 × 10^11^ CFU/g	*Lactiplantibacillus plantarum*	2.48	Yes
P3	Probiotic supplement	8.2 × 10^10^ CFU/g	*Lactiplantibacillus plantarum* *Lacticaseibacillus paracasei*	2.25 2.29	Yes
P4	Probiotic supplement	1.4 × 10^10^ CFU/g	*Lacticaseibacillus paracasei*	2.18	Yes
P5	Probiotic supplement	7.1 × 10^11^ CFU/g	*Lacticaseibacillus rhamnosus* *Lactiplantibacillus plantarum*	2.38 2.20	Yes
F1	Yogurt	3.1 × 10^8^ CFU/g	*Lactobacillus delbrueckii* subsp. *bulgaricus*	2.05	NI
F2	Yogurt	2.9 × 10^9^ CFU/g	*Lactobacillus delbrueckii* subsp. *bulgaricus*	2.30	NI
F3	Yogurt	2.6 × 10^8^ CFU/g	*Lactobacillus delbrueckii* subsp. *bulgaricus*	2.09	NI
F4	Yogurt	2.7 × 10^8^ CFU/g	*Lactobacillus acidophilus*	2.02	NI
F5	Yogurt	2.8 × 10^8^ CFU/g	*Lactobacillus acidophilus*	2.26	NI
F6	Yogurt	1.2 × 10^8^ CFU/g	*Lactobacillus delbrueckii* subsp. *bulgaricus*	2.16	NI
F7	Yogurt	3.4 × 10^7^ CFU/g	*Lactobacillus delbrueckii*	2.27	NI
F8	Yogurt	5.5 × 10^6^ CFU/g	*Lactobacillus acidophilus*	2.02	NI
F9	Organic cheese	7.52 × 10^8^ CFU/g	*Lactiplantibacillus plantarum* *Lactobacillus delbrueckii* subsp. *bulgaricus*	2.13 2.09	NI
F10	Organic cheese	4.2 × 10^6^ CFU/g	*Lactiplantibacillus plantarum*	2.28	NI
F11	Cheese	5.7 × 10^5^ CFU/g	*Lactiplantibacillus pentosus* *Lactiplantibacillus plantarum*	2.22 2.28	NI
F12	Cheese	3.9 × 10^5^ CFU/g	*Lactiplantibacillus plantarum* *Leuconostoc mesenteroides*	2.20 2.37	NI

(a) Results are expressed as means ± standard deviation (SD), with each data point being the average of two repeated measurements from a total of three independently replicated experiments: n = 3. The total plate count (CFU/g) was determined based on the bacterial isolation from the probiotic products during the initial isolation process, prior to any experimental treatments. The values represent the number of viable bacterial cells present in the product at the time of isolation. (b) The conformity of the product was evaluated if any information regarding the viable density or species identification was claimed. Yes, conformed to the indicated data; NI, no indication mentioned on the product. * Numbers following bacterial identities indicate the score value generated by Maldi-TOF. Scores ranging between 2.00 and 3.00 were attributed to “high-confidence identification” of species according to the manufacturer’s assessment.

**Table 2:** Acid and bile tolerance of isolated strains.

Origin	Strain ID	Species	Acid Tolerance			Bile Resistance		
			pH 2	pH 2.5	pH 3	0.3%	1%	2%
Probiotic Supplement	7M3	*L. acidophilus*	+	+	+	−	−	−
	7M2	*L. acidophilus*	+	+	+	+	+	+
	ATCC 8014	*L. plantarum*	+	+	+	+	+	−
	M16	*L. rhamnosus*	+	+	+	+	+	+
	Md3-5	*L. paracasei*	+	+	+	+	+	+
	M1	*Enterococcus faecium*	+	+	+	+	+	+
	M11	*L. helveticus*	+	+	+	+	+	+
	M5	*L. zeae*	+	+	+	+	+	+
	M9	*Enterococcus mundtii*	+	+	+	+	+	+
	51M	*L. fermentum*	+	+	+	+	+	+
	M33	*L. delbrueckii* subsp. *bulgaricus*	−	−	+	+	−	−
Yogurt	Y56	*L. delbrueckii* subsp. *bulgaricus*	+	+	+	+	+	+
	7M6	*L. rhamnosus*	−	−	+	+	−	−
Cheese	94m	*L. plantarum*	+	+	+	−	−	−
	45m	*L. delbrueckii* subsp. *bulgaricus*	+	+	+	+	−	−
	fC6	*Leuconostoc mesenteroides*	−	−	−	−	−	−
	M278	*L. pentosus*	+	+	+	+	+	+

**Table 3:** Antibiotic-susceptibility test results.

Origin	Strain	Species	Antibiotic Susceptibility		
			Erythromycin 15	Clindamycin 2	Gentamycin 10
Probiotic Supplement	7M3	*L. acidophilus*	+	+	+
	7M2	*L. acidophilus*	+	+	+
	ATCC 8014	*L. plantarum*	+	+	+
	M16	*L. rhamnosus*	−	+	+
	Md3-5	*L. paracasei*	+	+	+
	M1	*Enterococcus faecium*	+	+	+
	M11	*L. helveticus*	−	+	+
	M5	*L. zeae*	−	+	+
	M9	*Enterococcus mundtii*	+	+	+
	51M	*L. fermentum*	+	+	+
Yogurt	M33	*L. delbrueckii* subsp. *bulgaricuss*	+	−	−
	Y56	*L. delbrueckii* subsp. *bulgaricus*	−	+	+
	7M6	*L. rhamnosus*	+	+	+
Cheese	94m	*L. plantarum*	+	+	+
	45m	*L. delbrueckii* subsp. *bulgaricuss*	+	+	+
	fC6	*Leuconostoc mesenteroides*	+	+	+
	M278	*L. pentosus*	+	+	+

**Table 4:** Enzymatic (lipase, protease, and esterase) activities and hemolytic activity results.

Origin	Strain	Species	Hemolytic Activity	Protease Activity	Lipase Activity	Esterase Activity
Probiotic Supplement	7M3	*L. acidophilus*	γ-hemolysis	*+*	*−*	*−*
	7M2	*L. acidophilus*	γ-hemolysis	*+*	*−*	*−*
	ATCC 8014	*L. plantarum*	α-hemolysis	*+*	*−*	*−*
	M16	*L. rhamnosus*	γ-hemolysis	*−*	*−*	*−*
	Md3-5	*L. paracasei*	α-hemolysis	*+*	*−*	*−*
	M1	*Enterococcus faecium*	α-hemolysis	*−*	*+*	*+*
	M11	*L. helveticus*	γ-hemolysis	*−*	*−*	*−*
	M5	*L. rhamnosus*	γ-hemolysis	*+*	*−*	*−*
	M9	*Enterococcus mundtii*	γ-hemolysis	*−*	*−*	*−*
	51M	*L. fermentum*	α-hemolysis	*−*	*−*	*−*
Yogurt	M33	*L. delbrueckii* subsp. *bulgaricuss*	γ-hemolysis	*−*	*−*	*−*
	Y56	*L. delbrueckii* subsp. *bulgaricus*	γ-hemolysis	*−*	*−*	*−*
	7M6	*L. rhamnosus*	γ-hemolysis	*+*	*−*	*−*
cheese	94m	*L. plantarum*	γ-hemolysis	*−*	*−*	*−*
	45m	*L. delbrueckii* subsp. *bulgaricuss*	γ-hemolysis	*+*	*−*	*−*
	fC6	*Leuconostoc mesenteroides*	α-hemolysis	*−*	*+*	*+*
	M278	*L. pentosus*	γ-hemolysis	*+*	*−*	*−*

## Data Availability

The original contributions presented in this study are included in the article. Further inquiries can be directed to the corresponding author.
